# Sanitary Science

**Published:** 1860-05

**Authors:** 


☞ What Sanitary Science has done, and has yet to do, may be gathered from the following facts. The science is quite of modern date; but since the application of its simplest principles, the cleansing of streams, draining of houses, and introduction of pure water, the following evidence of benefits resulting has been given us:—In Liverpool the mortality had fallen from 37 in the 1000 to 27; in Bradford from 28½ to 22; in Gloucester, from 27 to 24. Taking an average of nineteen towns which had been treated in this way, it was found that the death-rate dropped from 28 in the 1000 to 21. Croyden was taken in hand scientifically some time ago; and since then an average of 196 lives have been saved in the town every year! The mortality among the pauper infants and pauper children in the metropolitan unions has been enormously reduced. In the Military School at Chelsea a death-rate of nine in the thousand has been brought down to one of four.

The female prisoners at Brixton, who live under sanitary rules, are three times as healthy as the poor needle-women of London; and at Pentonville, notwithstanding the allowance to be made for moral depression, the death-rate is only one-third of that prevailing in populous towns. But still there is a great work to be done; for as we are told, authoritatively, that at least 100,000 persons die annually in these islands at premature periods, and by preventible deaths; and at least 1,000,000 more are wasted and debilitated from similar causes. Talk of war, indeed! why what battles or contests ever wrought havoc like this—havoc, be it remembered, not occurring at intervals like an exceptional calamity, but carried steadily and incessantly through the ranks of our population? And we have still to remember the lesson taught us by the Crimean war. In that war we lost altogether 20,800 men; but of this number 5000 only were slain by the enemy. All the rest—15,800 soldiers—fell victims to privation and disease.—*London Medical Times & Gazette.*

